# MDSN: A Module Detection Method for Identifying High-Order Epistatic Interactions

**DOI:** 10.3390/genes13122403

**Published:** 2022-12-18

**Authors:** Yan Sun, Yijun Gu, Qianqian Ren, Yiting Li, Junliang Shang, Jin-Xing Liu, Boxin Guan

**Affiliations:** School of Computer Science, Qufu Normal University, Rizhao 276826, China

**Keywords:** high-order epistatic interactions, module detection, graph clustering, SNP network

## Abstract

Epistatic interactions are referred to as SNPs (single nucleotide polymorphisms) that affect disease development and trait expression nonlinearly, and hence identifying epistatic interactions plays a great role in explaining the pathogenesis and genetic heterogeneity of complex diseases. Many methods have been proposed for epistasis detection; nevertheless, they mainly focus on low-order epistatic interactions, two-order or three-order for instance, and often ignore high-order interactions due to computational burden. In this paper, a module detection method called MDSN is proposed for identifying high-order epistatic interactions. First, an SNP network is constructed by a construction strategy of interaction complementary, which consists of low-order SNP interactions that can be obtained from fast computations. Then, a node evaluation measure that integrates multi-topological features is proposed to improve the node expansion algorithm, where the importance of a node is comprehensively evaluated by the topological characteristics of the neighborhood. Finally, modules are detected in the constructed SNP network, which have high-order epistatic interactions associated with the disease. The MDSN was compared with four state-of-the-art methods on simulation datasets and a real Age-related Macular Degeneration dataset. The results demonstrate that MDSN has higher performance on detecting high-order interactions.

## 1. Introduction

With the gradual maturity of high-throughput sequencing technology, genome-wide association study (GWAS) has made considerable progress [1]. In the past few years, the GWAS has received extensive attention and obtained large amounts of research results. Although many complex diseases and traits have been proven to be related to a germline substitution of a single nucleotide at a specific position in the genome, more and more experiments further show that epistatic interaction is one of the important genetic bases for the occurrence and development of complex diseases [2]. Complex diseases are affected by a variety of genetic variations and environment factors. It is difficult for a single nucleotide polymorphism (SNP) to explain the genetic mechanism of complex disease states [3]. Studying the nonlinear interactions of SNPs, also known as epistatic interactions, plays a more important role in elucidating the genetic heterogeneity of complex diseases.

The study of epistatic interactions aims to find SNP interactions significantly associated with complex diseases and phenotypic defects at the genome-wide level. However, the huge amount of SNP genotype data brings great challenges to the study of genome-wide SNP interactions. High-dimensional genome-wide data mean that the identification of epistatic interactions is faced with a problem of combinatorial explosion. The key to solving this problem depends on how to find pathogenic interactions of SNPs from genome-wide data efficiently and effectively.

A simple and reliable exhaustive search method was published more than a decade ago. The exhaustive search method applied brute force cracking technique to the combinatorial search problem, enumerating all possible SNP interactions according to the preset association scale. Ritchie et al. [4] proposed MDR, which partitions the samples of a dataset into k-fold cross-validation groups to evaluate candidate interactions through a prediction model. The main advantages of MDR are parameter-free and facilitation, which is convenient for simultaneous detection and characterization of multiple SNPs. Ponte-Fernández et al. [5] proposed MPI3SNP, implementing a three-order exhaustive search for cluster topology through the cooperation of multi-CPU and multi-GPU clusters. In BOOST [6], Wang et al. designed a Boolean representation of genotype data achieving fast logic operation for the analysis of two-order SNP interactions in genome-wide data. The cost of detecting epistatic interactions is exponentially related to the order of the interactions to be considered. Hence, when dealing with the detection requirements of high-order epistatic interactions, the dimensional disaster and combinatorial explosion limit the exploration of high-order epistatic interaction studies by exhaustive methods. To speed up the identification of high-order epistatic interactions, search techniques such as filtering methods and random-search-based methods were proposed. Shang et al. [7] proposed a co-information theory-based method, EpiMiner, which is implemented in three stages for detecting epistatic interactions. EpiMiner has been applied to a real Age-related Macular Degeneration (AMD) dataset and captures important features in genetic architecture that have not been reported in the past. Liu et al. [8] proposed a flexible two-stage approach called HiSeeker to detect high-order epistatic interactions. HiSeeker makes use of a likelihood ratio test based on logistic regression to test and screen out the two-order SNP interactions related to the disease, and it is not sensitive to the marginal effects of a single SNP. Guo et al. [9] proposed a cloud computing technology-based algorithm DCHE, a key step in which is the dynamic clustering procedure for guiding how to merge genotype categories into a limited and variable number of groups. Its experiments on simulated datasets showed that DCHE has a considerable ability to detect interactions between two and three SNPs.

Swarm intelligence is a class of algorithms inspired by biological behavior, and it only needs to determine the representation of the problem, optimization function, and planning strategy to efficiently complete the task of exploring search space. The application of swarm intelligence optimization algorithms in high-order epistatic interaction detection has attracted wide attention [10,11,12,13]. MACOED [10] implemented a multi-objective optimization framework based on swarm intelligence optimization in the GWAS field, in which the framework implementation helps to increase the power and sensitivity. Sun et al. [14] proposed an algorithm IACO based on ant colony optimization and introduced a fitness function combining Bayesian networks and mutual information. Tuo et al. [11] proposed a niche harmony search method to detect high-order epistatic interactions associated with the phenotype. It utilized joint entropy as heuristic information to guide the search and selected two fitness scores to assess disease models.

The application of network science is permeating many fields, from mathematical science to life science. The module structure is not only an important feature of complex networks but also the organizational form of functional modules in biological complex networks. The biological network module detection method can systematically capture the interaction between genetic markers, and hence has become a powerful tool to find the pathogenic patterns of complex diseases. Bader et al. [15] proposed MCODE, which is the earliest protein complex detection method based on a seed node expansion strategy. MCODE constructs an effective module detection method and molecular interaction model to detect densely connected regions in the network. IG [16] designed a pairwise interaction detection method taking advantage of information gain, and then constructed an SNP interaction network, from which MCODE was applied to find modules that are regarded as high-order SNP interactions. Wang et al. [17] proposed a heuristic module detection method for searching protein complexes based on multiple topological features, which evaluates the weight of a node through clustering coefficient and node degree.

In this paper, a module detection method called MDSN is proposed for identifying high-order epistatic interactions. The construction of an SNP network is implemented by multi-order SNP interactions. The two-order and three-order SNP interactions with low mutual information values were filtered, and others were used for building the SNP network. Then, a node evaluation measure based on multi-topological features is proposed to improve the node expansion algorithm, where the importance of a node is comprehensively evaluated by the topological characteristics of the neighborhood. Finally, modules were detected in the SNP network, which are regarded as high-order SNP interactions associated with the disease.

## 2. Materials and Methods

**Figure 1:**
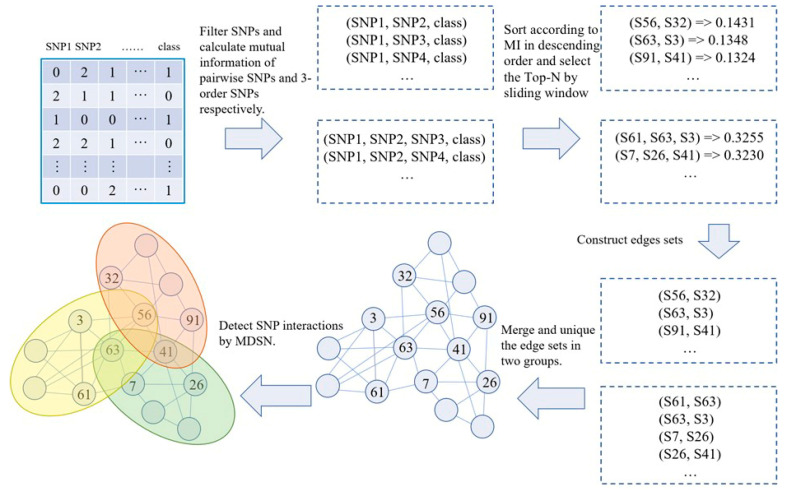
The MDSN flowchart. The numbers in the upper left corner of the table are the SNP data represented by real number coding, and each number in the lower left corner of the network represents the serial number of a SNP.

The MDSN includes two stages: the construction of the SNP network and the detection of SNP modules. In the stage of network construction, two-order and three-order SNP interactions are evaluated based on mutual information. A threshold selection strategy based on a sliding window is then used to filter SNP interactions. The selected SNP interactions are used to obtain edges to construct the SNP network. In the detection stage of SNP modules, the module mining strategy is designed to detect the SNP modules. Specifically, a module that initially includes only one seed node is extended recursively from its neighborhood nodes until no nodes can be selected to extend. The flowchart of the MDSN is in Figure 1.

### 2.1. Epistatic Interaction

Epistatic interactions are defined as phenotypic effects of multiple SNPs through nonlinear interactions based on population statistics. Identifying epistatic interactions and revealing their corresponding genes can further study the protein functions regulated by these genes and their genetic effects, which is one of the important ways to understand the pathogenesis of complex diseases.

The phenotype variable $Y=\left(y_{1},y_{2,}\ldots ,y_{J}\right)$ represents the disease status of *J* samples, including diseased samples and control samples. The variable $X=\left(x_{1},x_{2},\ldots ,x_{N}\right)$ represents the N SNPs in the dataset, and the element $x_{i}$ is an $SNP_{i}$ vector of length J. The SNP contains three genotypes, namely common homozygous genotype, heterozygous genotype and rare homozygous genotype, which are coded as 0, 1, and 2, respectively. For a *k*-order epistatic interaction, it can be evaluated by the measure of mutual information between the SNP set $X^{\prime }=\left(x_{1},x_{2},\ldots ,\;x_{k}\right)$, $\left(1<k<N,\;X^{\prime }\subseteq X\right)$ and the phenotype Y, which can be written as:

(1) $$MI\left(X^{\prime };Y\right)=H\left(X^{\prime }\right)+H\left(Y\right)-H\left(X^{\prime },Y\right)$$

where $H\left(X^{\prime }\right)$ is the entropy of $X^{\prime }$, H(Y) is the entropy of Y, and $H\left(X^{\prime },Y\right)$ is the joint entropy of $X^{\prime }$ and Y.

It is seen that epistatic interaction detection is a combinatorial optimization problem. However, it is impractical to evaluate all feasible SNP interactions; hence, the detection of high-order epistatic interactions remains a challenge. To address this complex problem, this paper proposed a module detection method, which can rapidly identify high-order epistatic interactions from a network science perspective.

### 2.2. SNP Network Construction

It has been widely accepted that epistatic interactions lead to the occurrence and evolution of complex diseases. Research on the causes of complex diseases is no longer limited to the detection of SNPs, but focuses on the detection of epistatic interactions [18]. SNP interactions are usually used to construct a network that depicts the topological relationships between SNPs. The method based on network modules can study biological functional networks and functional modules from the system level. In biological networks, there are usually modules with a tight local topology. The nodes in the module are more connected to other nodes inside the module than nodes outside it. The research on the module structure ignores the function of a single node, but focuses on exploring interactions of the nodes in the module [15,16,17]. In addition, modules are usually related to each other, and hence the neighborhoods that form a topological module may also exhibit similar or related functions.

MDSN obtains high-order epistatic interactions by identifying module structure in the network, instead of exhaustively testing all feasible SNP interactions. In the face of high-dimensional SNP data, the exhaustive computational cost of low-order (two-order and three-order) SNP interactions is affordable. However, when the detection target is a high-order epistatic interaction, the number of possible SNP interactions to be evaluated increases exponentially, and such a problem is difficult to solve with an exhaustive strategy. As a complex network, the SNP network has similar characteristics to the protein interaction network [19,20]. The SNP network also has significant modular characteristics.

Common SNP network usually consists of two-order SNP interactions. Nevertheless, two-order SNP interactions can easily form a star network with an SNP node showing strong main effect as the center, which is difficult to form a significant module structure. Inspired by the mining of complex network modules, MDSN adopts the strategy of multi-order SNP interactions complementing each other to construct a complex network, in which high-order epistatic interactions show a tightly connected module structure. Some two-order SNP interactions based on the mutual information measure can show strong interaction effects, while others have weak interaction effects or even none. Similarly, only some three-order SNP interactions show strong interaction effects. Hence, the MDSN uses both two-order and three-order SNP interactions with high mutual information values to construct the SNP network, and in it infers high-order epistatic interactions.

To avoid the dimension disaster, in this study, the multiSURF [21] method was used for SNP selection. Then, for all filtered SNPs, mutual information values of two-order and three-order SNP interactions were calculated, and a threshold selection strategy based on a sliding window was used to screen out SNP interactions with high mutual information values. Specifically, taking two-order as an example, all SNP interactions were sorted by their mutual information values in descending order, recorded as

S=[s(1),s(2),…, s(n)]] where *n* is the number of all SNP interactions, and s(i) is the mutual information value of *i*-th SNP interaction. Fluctuation score of s(i) is defined as

(2) $$Score\left(s\left(i\right)\right)=s_{i+2}-2\times s_{i+1}+s_{i}$$

Based on all fluctuation scores, their mean value (M) and variance (V), outliers can be captured by $\left\{s_{i}|Score\left(s_{i}\right)\notin \left[M-V,M+V\right]\right\}$. Finally, a sliding window with a preset window length was applied to slide from the left of fluctuation scores and stop when it cannot cover two outliers at the same time. The index of the midpoint of current window was considered as the threshold, and all SNP interactions with their indexes lower than the threshold were used for constructing SNP network. Similarly, the same strategy was used for three-order SNP interactions to select those with which to construct the SNP network.

All selected two-order and three-order SNP interactions were transformed into network edges. For a two-order SNP interaction, since it has strong interaction effect, it forms an edge between the related SNPs directly in the SNP network. For a three-order SNP interaction, such as (*SNP1*, *SNP2*, *SPN3*), it has strong interaction effect, and its 6 subsets usually, though not always, have strong interaction or marginal effects too. Based on this assumption, three edges, i.e., SNP1–SNP2, SNP1–SNP3, SNP2–SNP3, are added into the SNP network.

### 2.3. Module Detection

Functional module is an important topological feature of complex network, and SNP network resembles complex network and has similar characteristics. MDSN identifies high-order epistatic interactions by detecting functional modules in the SNP network, avoids evaluating a large number of feasible SNP interactions, and hence significantly reduces the running time. In the module detection stage, MDSN selects seed nodes first and then expands seed nodes to modules. For the selection of seed node, each node uses a measure based on multi-topological feature fusion to calculate the score, and the node with the highest score is selected as the seed node. The introduction of multi-topological feature fusion method can avoid the limitation of using only a single topological feature, which is usually insufficient to reflect the topological information of nodes in local subgraphs of complex networks.

The measure based on multi-topological feature fusion combines neighborhood density and node degree. The link between a node and its neighboring nodes can reflect the local topological characteristics of the subgraph where the node is located, while the node degree, a commonly used topology metric in network analysis, describes the number of links between a node and other nodes. The importance of a node can be inferred from the topology information of the subgraph where it is located. The weights of all nodes in the proposed method are calculated based on the node-link weights. Therefore, assigning weights to connections according to the structure of local subgraphs makes them have richer topology information, which is a key issue in this research.

HGCA [17] provides a good paradigm for evaluating node weights for multi-feature fusion. The iterative weighting strategy comprehensively considers the direct neighborhood of the node and the indirect neighborhood of a larger range, which make the calculation of the node weight more comprehensive. The calculation of the node weight takes the topological information of the connected edge into account. The weighting of the connected edges not only makes use of the node weight of the previous iteration, but also introduces the factor of node connectivity. Taking node $v_{i}$ as an example, its connectivity is defined as follows:

(3) $$C(v_{i)}=\frac{2\times \left|eN\left(v_{i}\right)\right|\times DN\left(v_{i}\right)}{|N\left(v_{i}\right)\times \left(\left|N\left(v_{i}\right)\right|-1\right)}$$

where $eN(v_{i})$ denotes the edge set of the subgraph $SG(v_{i})$, which is composed of node $v_{i}$ and its direct neighbor nodes. $DN\left(v_{i}\right)$ represents the maximum subgraph density of $SG(v_{i})$. $N(v_{i})$ represents the node set of the subgraph. The iterative calculation formula of node weight is as follows

(4) $$w^{t}\left(v_{i},v_{j}\right)=w^{t-1}\left(v_{i}\right)\times C\left(v_{i}\right)+w^{t-1}\left(v_{j}\right)\times C\left(v_{j}\right)+\underset{u\in N\left(v_{i}\right)\cap N\left(v_{j}\right)}{\sum }w^{t-1}\left(u\right)\times C\left(v_{u}\right)$$

(5) $$w^{t}\left(v_{i}\right)=\underset{v_{i}\in N\left(v_{i}\right)}{\sum }w^{t}\left(v_{i},v_{j}\right)$$

where $w^{0}\left(v_{i}\right)$ is initially set to 1, which means that the weight of each node in the network is the same at the beginning of the algorithm. The weight of each node is obtained through t rounds of iterative calculation, and the node with the largest weight is used as the seed node of the expansion operation.

After the seed node is determined, the node is regarded as an initial module, and then each direct neighbor node of the node is traversed and examined in turn. The selected seed node is initially regarded as a module structure containing only one node, and the nodes in the immediate neighborhood of the module are regarded as its candidate nodes. In the expansion process, nodes with high weight are added to the existing cluster, which is eventually expanded into a module. Then, the node with the next highest weight is selected as the seed node, and the above expansion process is repeated. The seed node expands into a stable module, the node with the second highest weight is selected as the seed node, and the above process is repeated until the termination condition is met. The extended modules are not removed from the network, and thus the algorithm can be regarded as an overlapping module detection method. In addition, according to the weight distribution of the nodes, the number of repetitions *N* of the module expansion operation is determined, and the module expansion contains a mode option on whether to over-prune or not.

## 3. Results

The performance of MDSN was compared with that of four state-of-the-art methods on the simulated dataset embedded with different-order disease models and a real AMD dataset. 

### 3.1. Evaluation Criteria

GAMETES [22] was used to generate the simulation data used in this experiment, with simulation models originating from Toxo [23]. The experiments in this section evaluate the performance of MDSN and comparison methods in terms of power and running time. Power reflects the detection effectiveness of methods in simulation experiments, evaluating the ability of these methods to accurately identify disease-causing SNP interactions from genetic data. The highest order of pathogenic interactions is eighth in the simulation data used. Although the proposed methods can output a set of solutions, it is difficult to accurately identify high-order pathogenic interactions. To comprehensively evaluate the performance of all methods, two evaluation measures with different emphases are used in this experiment. For the jth solution in a set of results, its accuracy is defined as:

(6) $$Acc\left(rel^{j}\right)=\frac{hit}{order}$$

where hit represents the number of SNP interactions that match the disease-causing SNPs in the corresponding simulation data, and order represents the order of the disease-causing interaction in the current simulation data. The evaluation of detection efficiency in the simulation experiment is divided into Power considering all the results, and Power considering only the optimal result. The definitions of the two powers are as follows:

(7) $$Power_{all}=\frac{\sum_{i=1}^{N}{\left[Acc\left(dat_{i}\right)\right]}_{avg}}{N}$$

(8) $$Power_{best}=\frac{\sum_{i=1}^{N}{\left[Acc\left(dat_{i}\right)\right]}_{max}}{N}$$

The data used in the simulation experiments include multiple sets of experimental data according to different parameters of various simulation models. Each dataset contains 30 simulation data generated by the same parameters of the same model. $dat_{i}$ represents the set of detection results obtained from the ith replicate dataset of simulation data by SNP interaction identification methods. The operator avg represents the average of the result set, and the operator max represents the maximum value of the result set.

The running time means the total elapsed time of the algorithm from the start of input of SNP genotype data to the termination of the run. The simulation experiments in this section compare the average running time of MDSN with the four comparison methods on each dataset.

### 3.2. Experimental on Simulated Data

For evaluating the MDSN method, 30 sets of simulation data with different types of various models were used on the simulation experiments. MDSN was compared with the four methods (FDHE-IW [24], EACO [25], EpiMOGA [13], and NHSA-DHSC [11]) in terms of power and runtime on simulation datasets with diverse heritability and model.

The simulation data contain six groups of SNP interaction pathogenic models of different orders and types. The model categories are multiplicative, additive, and threshold models, where the combined order of the model genotypes is from three to eight. In the multiplicative model, the prevalence of each genotype combination increases in a multiplicative manner, the prevalence of each genotype combination is the product of the effects within each locus, and the penetrance expressions of the model are all high-order polynomials. The genotype prevalence of the additive model is the sum of the pathogenic effects of each locus. The penetrance expression of the threshold model is a first-order polynomial. The number of SNPs in the simulated data is 1000, the sample size is 2000, and the sample is balanced. The heritability of the dataset was set to (0.05, 0.40). On the simulation data of different heritability and different orders, the power and running time of the five algorithms were compared. The parameters in MDSN were set to their default values, and all comparison methods were run using the parameters recommended at their publication.

**Figure 2:**
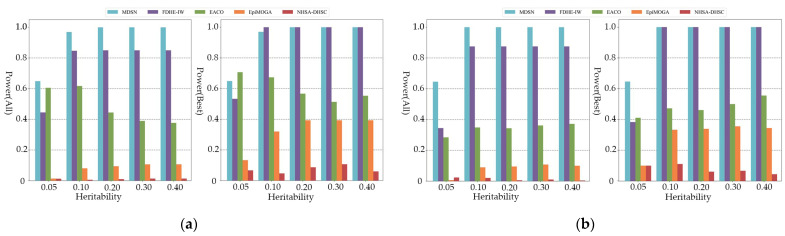
Power comparison on additive models: (**a**) Power comparison on five-order additive models; (**b**) Power comparison on six-order additive models.

**Figure 3:**
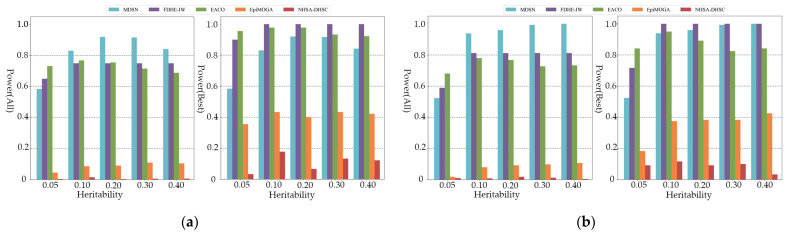
Power comparison on multiplicative models: (**a**) Power comparison on three-order multiplicative models; (**b**) Power comparison on four-order multiplicative models.

Figure 2 shows the power of all comparison algorithms on additive models of order five and six. As shown in the figure, MDSN and FDHE-IW have good performances on the simulation data of different heritability, and can accurately identify the pathogenic SNP model. When the performance evaluation only considers the best results, MDSN and FDHE-IW show similar detection ability. At a heritability of 0.05, the power of FDHE-IW dropped significantly and was less stable on datasets with low heritability. As shown in the experimental results of the multiplicative model in Figure 3, when the performance evaluation only considers the best results, the detection results of FDHE-IW reach the highest accuracy rate, and the performance of MDSN ranks second. EACO achieved a detection performance of about 0.8 on the three- and four-order simulation data, and the detection results can maintain a stable accuracy on data of different heritability. However, when the order of the pathogenic interactions in the simulation data reaches the five- and six-order, the performance of EACO decreases significantly.

**Figure 4:**
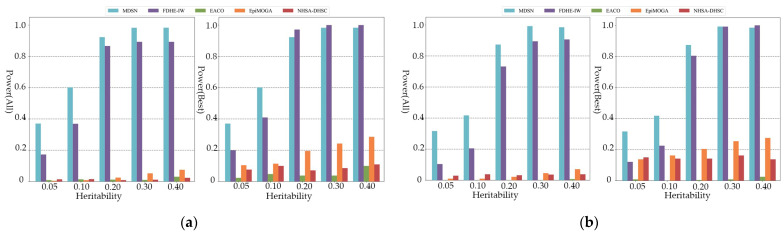
Power comparison on threshold models: (**a**) Power comparison on seven-order threshold models; (**b**) Power comparison on eight-order threshold models.

As shown in Figure 4, only MDSN and FDHE-IW can show the most effective detection ability when the order of the simulated pathogenic interaction reaches the seven and eight order. When considering all the results in the performance evaluation, the detection accuracy of EACO, EpiMOGA, and NHSA-DHSC are all below 0.08. From Figure 2, Figure 3 and Figure 4, we can see that in each group of experimental results, the detection performance of EACO, EpiMOGA, and NHSA-DHSC in the right figure is slightly improved compared with that in the left figure. This shows that the above algorithm can only provide a set of interactions with a high hit rate, and other interactions in the output result have a higher false-positive rate. EACO can perform well in low-order simulation data, but it is difficult to detect meaningful pathogenic interactions on seven- and eight-order data. Under different performance evaluation conditions, MDSN and FDHE-IW have the best or second-best performance on various types of simulation datasets.

Table 1 shows the running time of all methods on different simulation data. MDSN and FDHE-IW have similar performance on various simulation data, but FDHE-IW requires a huge time cost, and its running time can be a dozen times longer than that of MDSN. MDSN is an interaction pattern identification method based on module discovery. The running time is independent of the order of pathogenic interactions, and hence the proposed method can provide efficient detection performance when facing the application requirements of high-order interaction detection.

### 3.3. Experimental on Real AMD Data

To verify the effectiveness of MDSN on real disease data, this section conducts experiments using a real AMD dataset [26]. The AMD dataset is widely used in GWAS, which contains 96 cases, 50 control samples, and 103,611 SNPs. AMD refers to the degeneration of the macula in the elderly population, which leads to blurred vision and distortion of vision, and is an important cause of irreversible vision loss in the elderly. Precise identification of SNP interactions significantly associated with AMD can provide useful references for research in the diagnosis and treatment of this disease.

**Figure 5:**
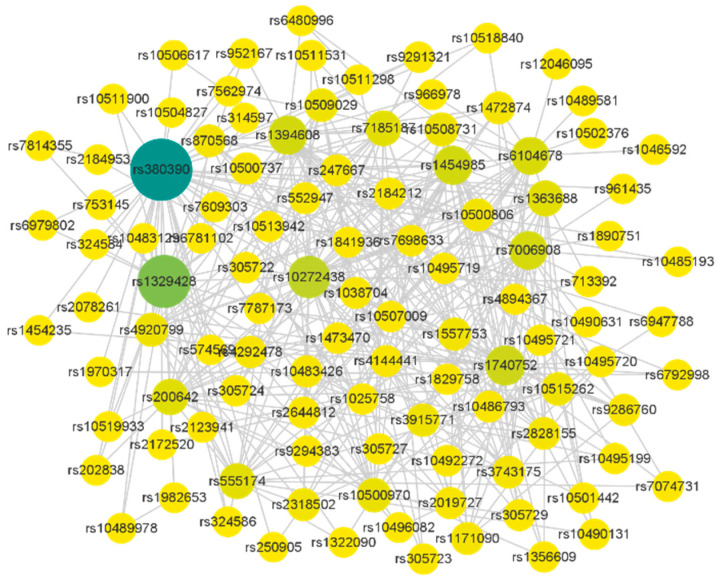
SNP interaction network constructed by MDSN. The color depth of the node indicates the difference of the node degree of the SNP, and the size of the node indicates whether the SNP has a known gene that can be mapped.

**Figure 6:**
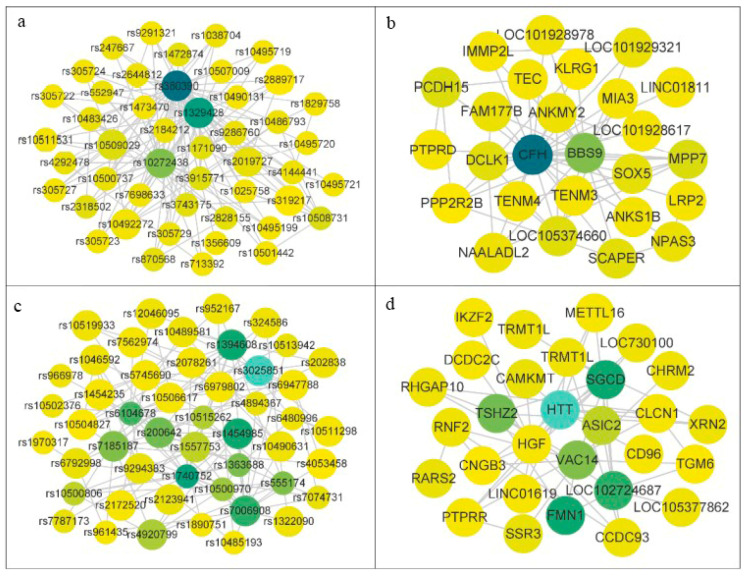
Visualization results of important community SNP networks and corresponding gene networks.

In this experiment, the missing values in AMD data are repaired according to the nearest neighbors of samples of the same class, and then the data are input to the MDSN algorithm after repair. MDSN is applied to the SNP network constructed by the AMD dataset, which is constructed by a multi-association interaction complementary method, as shown in Figure 5. Table 2 shows the SNP communities detected by MDSN, Figure 6 shows the visualization results of important module SNP networks and corresponding gene networks, and Table 3 shows the SNPs associated with AMD that have been validated by relevant studies.

On the AMD dataset, some SNP interactions significantly associated with AMD were identified by MDSN. As shown in Table 2, the SNP interaction modules obtained by MDSN are two eight-order modules, one ten-order module, and one eleven-order module. The table shows the SNPs and the genes included in the module. Among the SNPs in the module, rs1329428 and rs2019727 reside in the *CFH* gene on human chromosome 1. Both of these two SNPs have been proved by biological experiments to increase the risk of AMD [26,27,28,29]. They have also been reported to be associated with plasma *CFH* or *CFHR1* concentration in the GWAS. SNP rs319217 is located on the *PPP2R2B* gene on chromosome 1. Abnormal expression of the *PPP2R2B* gene leads to spinocerebellar ataxia, which leads to a gradual weakening of eye movement coordination in patients [30,31]. SNP rs10504827 is located on the *CNGB3* gene on human chromosome 8, and mutations in this gene cause macular and cone–rod dystrophy [32,33]. SNP rs1329428 is widely recognized as a mutation associated with AMD pathogenesis, and Ansari et al. [29] demonstrated that rs2019727 increases the risk of AMD. rs10504827 and rs319217 can be queried in the Gene Card database for their association with AMD disease. In addition to the above genes related to AMD, other genes shown in Table 2 also have potential biological correlation with other diseases. SNP rs1046592 is located on the *RNF2* gene [34]. *RNF2* is the core subunit of *PRC1*, which is a negative regulator of anti-tumor immunity in various human cancers, including breast cancer. Studies have shown that the expression of *RNF2* is related to the decrease in cytotoxicity of tumor infiltrating immune cells. SNP rs2250886 is located on the *DNHD1* gene [35]. Studies have shown that *DNHD1* mutation can lead to sperm motility deficiency. This discovery provides important insights into the biological basis of this disease, and helps to consult the affected individuals. SNP rs12046095 is located in the *TRMT1L* gene, which is involved in cognitive function. In fact, knocking out *TRMT1L* in mice has been proved to lead to changes in motor coordination and abnormal exploratory behavior, indicating that its activity is related to neurological function [36].

**Table 2:** Results obtained by MDSN and the genes involved. The underlined SNP is the AMD-related SNP with strong main effect detected by MDSN.

Module	SNPs	Gene
1	rs6114139, rs1046592, rs2250886, rs1683 147, rs7609303, rs305723, rs1329428, rs12046095	*RNF2, DNHD1, CFH, TRMT1L*
2	rs1046592, rs2250886, rs1683147, rs7609303, rs305723, rs1329428, rs1924257, rs12046095	*RNF2, DNHD1, CFH, TRMTIL, LOC107985255*
3	rs1046592, rs2250886, rs1683147, rs7609303, rs305723, rs1329428, rs319217, rs6467309, rs2019727, rs12046095	*RNF2, DNHD1, CFH, TRMT1L, PPP2R2B, COPG2*
4	rs6114139, rs1046592, rs2250886, rs1683147, rs7609303, rs305723, rs1329428, rs10504827, rs6467309, rs2019727, rs12046095	*RNF2, DNHD1, CFH, TRMT1L, CNGB3, COPG2*

**Table 3:** SNPs associated with AMD in detected modules.

SNP	Gene	Chromosome	References
rs1329428	*CFH*	1	[7,24]
rs2019727	*CFH*	1	[26,27]
rs10504827	*CNGB3*	8	[30,31]
rs319217	*PPP2R2B*	5	[28,29]

## 4. Discussion

In this work, we propose a module detection based method MDSN for identifying high-order epistatic interactions at the genome-wide level. MDSN includes the two stages of SNP interaction network construction and network module detection. In the interaction network construction stage, we adopt a multi-order interactions complementary strategy to construct the network. The two-order and three-order SNP interactions together provide network association information. The two-order interaction constitutes the basic topology of the interactive network. Complementing the combinatorial effect, the three-order combinatorial increases the connectivity between nodes, resulting in a tighter modular structure of the network. The improved seed node expansion algorithm is applied to the module detection of the SNP interaction network.

To verify the performance of MDSN, we conducted experiments on simulated and real datasets, respectively. In the simulated experiment, we compared MDSN with four state-of-the-art swarm intelligence algorithms on six different models. The performance of the high-order datasets in the simulated experiment shows that MDSN is promising for the detection of high-order SNP interactions. In the real AMD data, most of the SNP interactions we detected have been confirmed to be associated with the AMD disease. The above experiments show that MDSN is an effective method for detecting high-order epistatic interactions.

## 5. Conclusions

MDSN is a new method that can effectively solve the combinatorial explosion problem of high-order epistatic interaction detection. From the perspective of the network, this method searches for high-order SNP interaction patterns using the network module detection. Due to the high computational cost, it is difficult for combinatorial search methods to efficiently identify higher-order interactions in the high-dimensional space. Therefore, compared to combinatorial search methods represented by swarm intelligence, module mining in SNP interaction networks has greater advantages in making full use of the biological network topology information constructed by biomarker associations. The source code of MDSN is available on the GitHub repository: https://github.com/CDMB-lab/MDSN (accessed on 5 December 2022).

Simulation experimental results show that MDSN is superior to other comparative methods. For the detected SNP modules, some of them can be confirmed to be AMD-related. However, due to the lack of effective biological verification experiments, it is difficult to give detailed biological explanations for the detection results of real disease data. Therefore, detecting more high-order epistatic interactions that can be demonstrated to correlate with disease data is the direction of our future research.

## Figures and Tables

**Table 1:** Mean runtime of methods on simulation models (unit: seconds).

Model	MDSN	FDHE-IW	EACO	EpiMOGA	NHSA-DHSC
Additive-5	36.64	298.46	246.19	416.47	66.01
Additive-6	37.60	691.08	241.08	454.04	66.28
Multiplicative-3	37.47	49.88	241.45	296.80	76.75
Multiplicative-4	36.96	124.20	241.36	374.74	68.89
Threshold-7	39.02	1375.88	241.47	487.88	66.50
Threshold-8	38.94	2875.88	241.59	513.62	92.53

## Data Availability

Not applicable.
